# Transcriptome Signature of Immature and In Vitro-Matured Equine Cumulus–Oocytes Complex

**DOI:** 10.3390/ijms241813718

**Published:** 2023-09-06

**Authors:** Alejandro de la Fuente, Charles Scoggin, Etta Bradecamp, Soledad Martin-Pelaez, Machteld van Heule, Mats Troedsson, Peter Daels, Stuart Meyers, Pouya Dini

**Affiliations:** 1 Department of Anatomy, Physiology and Cell Biology, School of Veterinary Medicine, University of California, Davis, CA 95616, USA; 2 Department of Population Health and Reproduction, School of Veterinary Medicine, University of California, Davis, CA 95616, USA; 3 LeBlanc Reproduction Center, Rood and Riddle Equine Hospital, Lexington, KY 40511, USA; 4 Department of Morphology, Imaging, Orthopedics, Rehabilitation and Nutrition, Faculty of Veterinary Medicine, University of Ghent, 9820 Merelbeke, Belgium; 5 Gluck Equine Research Center, University of Kentucky, Lexington, KY 40506, USA

**Keywords:** oocyte, cumulus cell, in vitro embryo production, in vitro oocyte maturation, equine, transcriptome

## Abstract

Maturation is a critical step in the development of an oocyte, and it is during this time that the oocyte advances to metaphase II (MII) of the meiotic cycle and acquires developmental competence to be fertilized and become an embryo. However, in vitro maturation (IVM) remains one of the limiting steps in the in vitro production of embryos (IVP), with a variable percentage of oocytes reaching the MII stage and unpredictable levels of developmental competence. Understanding the dynamics of oocyte maturation is essential for the optimization of IVM culture conditions and subsequent IVP outcomes. Thus, the aim of this study was to elucidate the transcriptome dynamics of oocyte maturation by comparing transcriptomic changes during in vitro maturation in both oocytes and their surrounding cumulus cells. Cumulus–oocyte complexes were obtained from antral follicles and divided into two groups: immature and in vitro-matured (MII). RNA was extracted separately from oocytes (OC) and cumulus cells (CC), followed by library preparation and RNA sequencing. A total of 13,918 gene transcripts were identified in OC, with 538 differentially expressed genes (DEG) between immature OC and in vitro-matured OC. In CC, 13,104 genes were expressed with 871 DEG. Gene ontology (GO) analysis showed an association between the DEGs and pathways relating to nuclear maturation in OC and GTPase activity, extracellular matrix organization, and collagen trimers in CC. Additionally, the follicle-stimulating hormone receptor gene (*FSHR*) and luteinizing hormone/choriogonadotropin receptor gene (*LHCGR*) showed differential expressions between CC-MII and immature CC samples. Overall, these results serve as a foundation to further investigate the biological pathways relevant to oocyte maturation in horses and pave the road to improve the IVP outcomes and the overall clinical management of equine assisted reproductive technologies (ART).

## 1. Introduction

In vitro embryo production (IVP) is a rapidly expanding field in the equine breeding industry, increasing the efficiency of breeding programs [1] and contributing to the production of live foals from valuable mares and stallions which could not otherwise produce progeny using traditional breeding methods [2]. The retrieval of immature oocytes from antral follicles of varying sizes and developmental stages from standing sedated mares is an important source of female gametes for IVP. Immature oocytes with surrounding cumulus cells, also known as cumulus–oocyte complexes (COC), need to be matured in vitro to initiate the resumption of meiosis and acquire fertilization and developmental competence before they can be fertilized and undergo embryogenesis. Current maturation rates are variable among laboratories, with reported maturation rates averaging ~65% [3,4,5,6,7]. Additionally, in vitro-matured oocytes show lower embryo development rates compared to in vivo-maturing oocytes [8].

During maturation, changes occur in the nucleus and the ooplasm of the oocyte, which have been defined as nuclear maturation and cytoplasmic maturation, respectively [9]. Nuclear maturation involves the resumption of the first meiotic division, starting from the germinal vesicle stage (Diplotene stage of Prophase I) and arresting in metaphase II of the second meiotic division, resulting in the extrusion of the first polar body [10,11,12,13]. In the follicle, there is an association between the rise of the luteinizing hormone (LH) and the resumption of oocyte maturation [14]. However, the specific physiological signaling cascade caused by gonadotropins during equine in vitro maturation (IVM) is unknown, and it remains unclear whether alternative pathways are activated for the resumption of meiosis compared to in vivo maturation.

Nuclear maturation is the most widely used method to classify an oocyte as mature, and it is determined by the presence of the first polar body in the perivitelline space between the oocyte and the zona pellucida. Simultaneously, cytoplasmic maturation occurs and is characterized by changes in the ooplasm, including organelle redistribution and the reorganization of the vesicles, mitochondria, Golgi apparatus, endoplasmic reticulum, and cortical granules in the process referred to as cytoplasmic maturation [15,16]. These modifications are considered key events that facilitate monospermic fertilization and determine the ability of the oocyte to become a blastocyst [15,17]. The changes in the nucleus and the ooplasm are the results of several signaling pathways within a COC with an orchestrated regulatory loop between the cumulus cells and the oocyte [18,19]. In addition to the resumption of meiosis, gonadotropins also act on cumulus cells, stimulating the expansion of the extracellular matrix through the synthesis of hyaluronic acid, which is another sign of oocyte maturation [20].

Cumulus cells control oocyte maturation by maintaining meiotic arrest [21] at first, followed by inducing meiotic resumption [20], and by regulating changes in the ooplasm for the developmental competence of the embryo after fertilization [22]. This dynamic control of cumulus cells over the oocyte suggests that the transcriptome systematically changes throughout maturation, presenting unique molecular signatures during the maturation process [23]. Transcriptomic studies have shown that the gene expression profile of human cumulus cells varies based on the degree of nuclear maturation and maturation conditions [24,25]. Moreover, it has been shown that oocytes show distinct transcriptomes at different maturation stages [26]. Overall, these studies reveal a complex gene network, including genes associated with gonadotropin receptors and the TGF-Beta family in oocyte maturation. However, the molecular signature of equine oocytes and cumulus cells and their crosstalk during maturation has not yet been described. Despite ongoing research on equine oocyte IVM, there is a gap in our understanding of this dynamic process. Hence, the objective of this study was to characterize the transcriptome profile of immature and in vitro-matured equine oocytes and their surrounding cumulus cells.

## 2. Results

To elucidate transcriptional changes between the immature and in vitro-matured oocytes, COCs were collected via transvaginal aspiration. RNA was extracted from both the oocytes (OC) and their surrounding cumulus cells (CC), followed by library preparation and RNA sequencing (refers to Section 4).

### 2.1. Transcriptome Profile of Equine Oocytes and Cumulus Cell

On average, ~80 million raw reads were generated from each sample, with >90% demonstrating quality over Q30 (Supplementary Table S1). There was no difference between the amount of generated data, quality, and the percentage of guanine–cytosine (GC) content among the groups (*p* > 0.05). Data were mapped to the equine reference genome (EquCab3.0) with an average of a 95% mapping rate (range: 93.4–95.9%; Supplementary Table S1).

**Figure 1:**
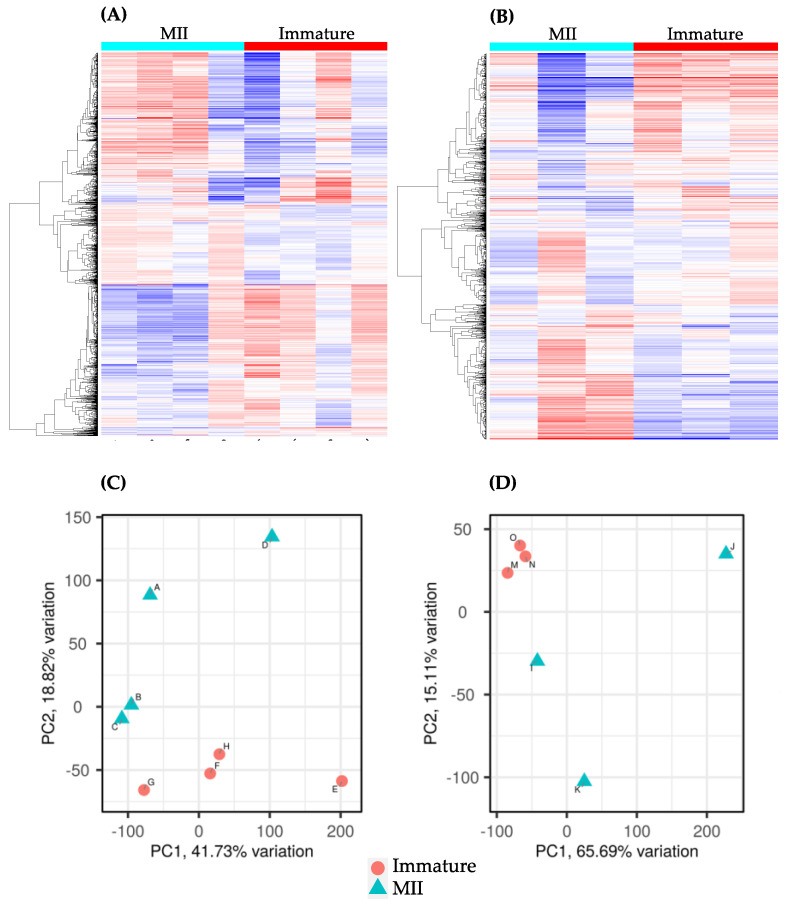
Gene expression patterns of mature and immature oocytes and cumulus cells. Heat map graphs comparing the immature group to the in vitro-matured group (MII) in OC (**A**) and CC (**B**) samples. Samples are in columns, and the 1500 most variable genes are in rows. Expression is indicated by the z-score in a colorimetric scale (blue: −4 to red +4). The principal component analysis (PCA) plots of OC samples (**C**) (immature samples E, F, G, H; MII samples A, B, C, D) and CC samples (**D**) (immature samples M, N, O); MII samples I, J, K) show the samples distributed in the graph based on their gene expression similarities. Samples appear to show separation between PC1 and PC2 by maturity status in both types of samples.

In OC, 13,918 gene transcripts were detected, with 68.46% categorized as protein-coding genes (Supplementary Table S2). In CC, 13,104 gene transcripts were detected, with 68.47% categorized as protein-coding genes (Supplementary Table S3). Gene expressions across OC and CC samples are presented in a heat map (Figure 1A and Figure 1B, respectively). Based on the principal component analysis (PCA), individual samples were clustered based on their corresponding groups (Figure 1C and Figure 1D, respectively).

### 2.2. Dynamics of Gene Expression during Oocyte Maturation

**Figure 2:**
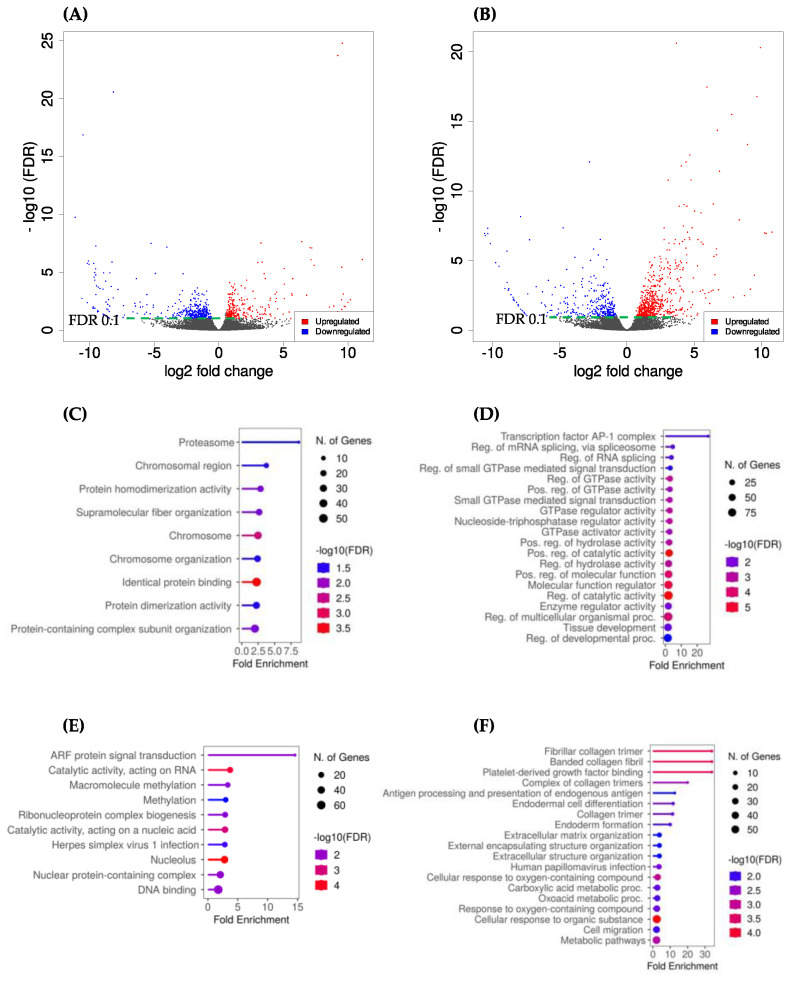
Differentially expressed genes (DEG) represented in a volcano plot for oocyte samples (**A**) and CC samples (**B**) denoting the downregulated genes (blue) on the left compared to upregulated genes (red) on the right using a fold change cutoff of 1.5. The dotted green line indicates the FDR cutoff of 0.1. GO analysis for enriched pathways of genes highly expressed in OC-MII (**C**) and lowly expressed in OC-MII (**E**) relative to immature OC, and those highly expressed within in vitro matured CC (**D**) and those lowly expressed within in vitro CC (**F**) relative to immature CC.

In OC, comparing DEGs between immature OC and in vitro-matured OC (OC-MII) revealed 538 DEGs with 180 upregulated and 358 downregulated genes in the OC-MII group relative to the immature OC group (Supplementary Table S4, Figure 2A). The gene ontology analysis revealed that pathways associated with upregulated genes were related to aspects of nuclear maturation, such as chromosome organization, the DNA packaging complex, and the centrosome, while downregulated genes were related to nucleolus and ribonucleoprotein complex biogenesis. (Figure 2C and Figure 2E, respectively).

In CC, 871 DEGs were identified, with 569 upregulated and 302 downregulated genes in the in vitro-matured CC (CC-MII) group relative to the immature CC group (Supplementary Table S5, Figure 2B). Based on the gene ontology analysis of DEGs, the most relevant pathways associated with upregulated genes were related to GTPase activity (Figure 2D), while pathways associated with downregulated genes were mostly related to extracellular space, extracellular matrix organization, and collagen trimers (Figure 2F).

### 2.3. Gonadotropin, Aromatase, and Extracellular Remodeling-Related Genes

Among the DEGs, the LH-choriogonadotropin receptor gene (*LHCGR*) showed a lower expression (log2FC: −2.54, FDR: 0.00979) within in vitro-matured CC compared to immature CC, while the follicle-stimulating hormone (FSH) receptor gene (*FSHR*) showed a higher expression (log2FC: 3.3 FDR: 0.00123) in the CC-MII samples compared to immature CC. The cytochrome P450 aromatase gene (*CYP19A1*) (log2FC: 6.882; FDR: 3.78 × 10^−12^) and the Estradiol 17-beta-dehydrogenase gene (*HSD17B1*) (log2FC: 6.719; FDR: 4.28 × 10^−15^) were both upregulated in CC-MII samples.

Several genes associated with extracellular space and a collagen trimer were found to be differentially expressed in CCs. Collagen type I alpha 1 chain (*COL1A1*; log2FC: −3.51; FDR: 0.0644), collagen type I alpha 2 chain (*COL1A2*; log2FC: −3.36; FDR: 0.00052), collagen type III alpha 1 chain (*COL3A1*; log2FC: −3.99; FDR: 0.00318), collagen type V alpha 1 chain (*COL5A1*; log2FC: −2.64; FDR: 0.00702), collagen type VI alpha 1 chain (*COL6A1*; log2FC: −4.07; FDR: 0.0306), collagen type XIV alpha 1 chain (*COL14A1*; log2FC: −4.44; FDR: 0.00447), and collagen type XVI alpha 1 chain (*COL16A1*; log2FC: −2.21; FDR: 0.0257) were downregulated in CC-MII samples, while only collagen type XI alpha 1 chain (*COL11A1*; log2FC: 2.1; FDR: 0.0178) showed an upregulation in CC-MII samples.

### 2.4. Oocyte and Cumulus Cell Crosstalk- Ligand–Receptor Interaction Analysis

**Figure 3:**
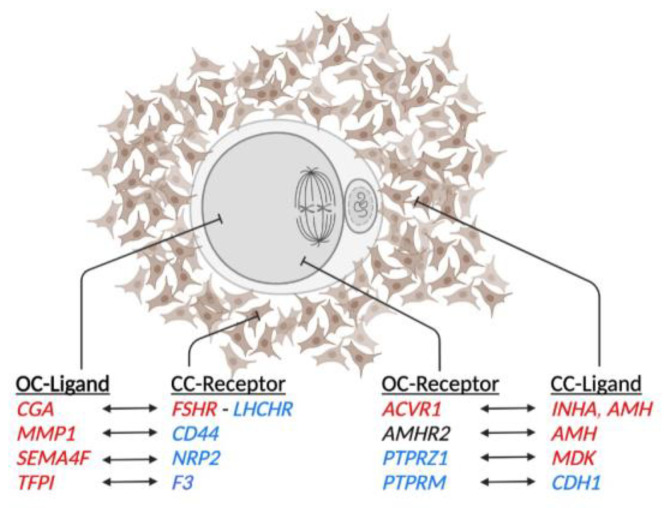
Ligand–receptor pairs between oocytes and cumulus cells. Schematic representation of the suggested crosstalk between oocyte and cumulus cells based on DEG between immature and in vitro-matured samples. Genes are colored based on their expression: red represents high expression, and blue represents a low expression of genes in MII samples relative to immature samples. Genes in black correspond to those expressed but not differentially expressed. Illustration made in BioRender.com.

Differentially expressed genes in both OC and CC samples were used to identify ligand–receptor pairs described in the FANTOM5 database. In the first step, genes identified as a ligand in OC and those identified as receptors in CC were used to construct pairs. Then, genes identified as receptors in OC and as ligands in CC were also noted (Table 1). Among these potential communication paths, the anti-Müllerian hormone gene (*AMH*) showed a higher expression (log2FC: 3.02; FDR: 0.0324) in CC-MII compared to immature CC. It is worth noting that the AMH receptor 2 gene (*AMHR2*) was expressed in OC and CC. The inhibin α subunit gene (*INHA*) presented a higher expression (log2FC: 2.1; FDR: 0.0168) in the CC-MII group compared to the immature CC, while there was no difference in the expression level of the inhibin subunit β-A gene (*INHBA*) between immature CC and CC-MII. Activin A receptor type I (*ACVR1*) showed a high expression (log2FC: 1.2; FDR: 0.0865) in OC-MII relative to immature OC. The glycoprotein hormone α polypeptide (*CGA*; the α chain segment of gonadotropin dimers) gene showed a higher expression (log2FC: 4.39; FDR: 0.00341) in OC-MII compared to immature OC. Nevertheless, a high expression of *FSHR* and a low expression of *LHCGR* were observed within in vitro CC, as mentioned above. A graphical representation of the suggested ligand–receptor interactions is presented in Figure 3.

## 3. Discussion

During the final stages of oogenesis, the oocyte acquires meiotic and developmental competence in a process known as oocyte maturation [27]. Oocyte maturation is a strictly regulated process that occurs within the follicle. However, with the implementation of ART and retrieval of immature oocytes, this process must occur in laboratory settings that try to simulate the in vivo conditions. In part, due to the lack of a full understanding of the dynamics of oocyte maturation in the horse, IVM results in suboptimal outcomes that also affect IVP.

The results of the present study show that a relatively similar number of genes are expressed in oocytes (13,918) and cumulus cells (13,104) for the 20,322 protein-coding genes found in the horse genome [28]. Based on our data and observed in the heatmaps and PCA plots, a characteristic gene expression pattern was observed among immature and in vitro-matured OC and CC samples. OC samples appeared to be scattered, but both groups were separated from each other. On the other hand, immature CC samples formed a defined cluster, while in vitro matured CC samples were relatively scattered. It is important to mention that oocytes in this study were classified as mature based on the presence of a polar body, which is the accepted standard assessment of nuclear maturation in an IVP setting. Therefore, it is possible that the lack of uniformity of gene expression among the matured samples is a reflection of the different levels of cytoplasmic maturity among OC-MII samples at the time of nuclear maturation assessment. Even though some aspects of cytoplasmic maturation require the signaling provided by the release of germinal vesicle content [29], the signaling mechanisms regulating nuclear and cytoplasmic maturation are not necessarily completely coordinated [30]. Thus, a non-invasive assay that could serve to evaluate the oocyte’s cytoplasmic maturation in addition to its nuclear maturation status could better predict the developmental competence of the oocyte.

Following the analysis of enriched pathways among the DEGs in OC, it was determined that genes with a higher expression in matured OC samples were related to nuclear maturation, including pathways of the chromosomal region, centrosome, chromosome organization, and sister chromatid cohesion. An important event that occurs during oocyte maturation is the resumption of meiosis, restarting from an arrested oocyte at prophase I and advancing to the metaphase II stage, where the oocyte is arrested again until fertilization occurs. However, the mechanism regulating the meiotic cycle in equine oocytes has yet to be determined. A highly elaborated mechanism has been described in detail for other species [31,32,33]. In short, it has been shown that the maturation promotor factor (MPF) is the complex that drives the meiotic cycle in oocytes. The MPF is composed of two main subunits: cell division cycle 2 (CDC2), also known as cyclin-dependent kinase 1 (CDK1) [34], and the regulatory subunit cyclin B1 (CCNB1) [35]. Meiotic arrest at prophase I is maintained by high levels of cGMP that diffuse into the oocyte from the surrounding cells through gap junctions. cGMP inhibits cyclic nucleotide phosphodiesterases (PDE), maintaining high levels of cAMP within the oocyte that act on cell cycle molecules maintaining meiotic arrest [36]. The effect of gonadotropins, particularly LH, has been described as one of the triggering signals to resume meiosis. Gonadotropins cause a decrease in cGMP in the oocyte via two main pathways. The first pathway is the phosphorylation of connexin 43 (gap junction α 1 [GJA1]), one of the main structural molecules of gap junctions between cumulus cells and the oocyte, thus hampering the transportation of cGMP to the oocyte [37]. In this study, it was observed that *GJA1* was expressed in immature CC and CC-MII but was not differentially expressed between the groups. This observation coincides with a previous study using the reverse transcription–polymerase chain reaction (RT-PCR) with specific primers, which determined that connexin 43 mRNA levels did not change during IVM [38], and the same was observed at the protein level using gel electrophoresis and immunoblotting [39]. Connexin 37 (gap junction α 4 [GJA4]) is another structural protein of gap junctions and is found within the plaque between cumulus cells and the oocyte [40,41]. In this study, *GJA4* was found to be upregulated in CC-MII compared to immature CC, while it had a similar expression pattern between immature OC and OC-MII. It was expected that connexin 37 was reduced in the mature oocytes; thus, the observed upregulation of *GJA4* could be associated with an alternative maturation pathway in vitro. One of the limiting points in our study is the inclusion of only two stages (immature and MII). Analyzing the CC at different times during maturation could further elucidate the expression pattern of this gap junction. Overall, the specific significance of these results in the communication between the oocyte and cumulus cells needs to be determined in future studies.

The second pathway that leads to a decrease in cGMP in the oocyte is the reduction in the levels of cGMP through the activation of cGMP-specific 3′,5′-cyclic phosphodiesterase (PDE5) [42,43]. Other molecules implicated in the regulatory mechanism of meiosis are the cyclin-like DNA glycosylase cyclin O (CCNO) and the cell division cycle-6 homolog (CDC6). The former has been described in mice as a regulator of the resumption of meiosis, and the formation of microtubule organization centers (MTOCs) [11] and the latter has been proposed as a regulatory molecule during the organization of microtubules during centrosome formation [44]. In the present study, there was no difference in the expression of *CDK1* or *CCNB1* between immature OC and OC-MII samples; however, *CCNO* and *CDC6* were downregulated in OC-MII samples. We also observed that *PDE7A*, a cAMP-specific phosphodiesterase, was upregulated in OC-MII samples along with *PDE5A*. While in CC-MII samples, the cGMP-specific phosphodiesterase *PDE6D* was upregulated, *PDE3B* and *PDE7A* were downregulated. These changes suggest the activation of downstream cascades associated with the resumption of the meiotic cycle, presumably due to the gonadotropin present in the maturation medium. However, the complete upstream regulators need to be identified. The meiotic cycle progresses through a series of arrest and resumption steps during the maturation process. Therefore, the interpretation of the transcriptome of all the genes related to the meiotic cycle was limited in this study and insufficient to determine the dynamics of the meiotic cycle regulation in horses. More detailed studies are necessary to understand the molecules driving the resumption of meiosis in the equine oocyte during maturation. Of note, one of our study’s limitations is the absence of protein analysis to complement our transcriptomic approach and identify changes in CC and OC during maturation. However, by examining transcriptomic changes, we provide an insight into the crucial modifications occurring during IVM.

In this study, upregulated genes in CC-MII were associated with GTPase activity. GTPases are hydrolase enzymes that act independently by hydrolyzing GTP to GDP in the cytoplasm. Members of the Rho and Ran subfamilies of small GTPases are largely associated with cytoskeletal functions in cell organization and movement by regulating actin and microtubule filaments [45]. In porcine oocytes, the small Rho GTPase (RhoA) has a key role in regulating the organization of the cytoskeleton, including cell migration, polarity, and division [46]. In the present study, it was found that Ras homolog family member B (*RHOB*) had a higher expression in CC-MII samples compared to immature CC. Moreover, Ran GTPase activating protein 1 (*RANGAP1*) showed a lower expression (log2FC: −1.22; FDR: 4.73 × 10^−2^) in CC-MII samples. Even though the GO analysis did not reveal GTPase pathways in OC samples, it was found that *RHOB* (log2FC: −1.91; FDR: 2.35 × 10^−2^) and *RANGAP1* (log2FC: −1.7; FDR: 4.92 × 10^−2^) were downregulated in OC-MII samples. The genes responsible for cytoskeletal and cell organization are necessary for the correct completion of cytoplasmic maturation as there is a reorganization of organelles; thus, determining the main molecules in horses could help us understand the variation in developmental competence among nuclear-matured oocytes.

Another finding in the present study was that *LHCGR* presented a low expression in CC-MII samples relative to immature CC. It has been reported that the expression of *LHCGR* on porcine CC depends on oocyte factors as well as the levels of FSH available in the culture media [47,48]. In the present study, a commercial maturation medium was used and its proprietary information about gonatropic hormones and their concentration was not disclosed. Nevertheless, based on preliminary data obtained from a parallel study aiming to compare the effect of different maturation media on the gene expression of equine CC (de la Fuente and Dini, unpublished data), it was confirmed that the expression of *LHCGR* is comparatively low in mature relative to immature samples in all tested media. Similar to the current study, it was also observed that *FSHR* presented a higher expression in CC-MII samples relative to immature CC regardless of the maturation media (de la Fuente and Dini, unpublished data). It has been previously reported that *FSHR* is expressed in cumulus cells from immature and in vitro-matured COCs [38]. It is also known that binding LH and FSH to their receptors leads to a cascade of pathways, including changes in the cAMP and cGMP concentration in vitro, leading to the resumption of meiosis in murine and human oocytes [49]. FSHR is a G-protein-coupled receptor with seven transmembrane domains, and it has been shown to activate the classical FSHR/Adenylyl cyclase (AC)/cAMP/protein kinase A (PKA) pathway [50]. When FSH is present in an in vitro maturation medium, it has the capacity to initiate oocyte meiotic resumption in mice [51], porcine [52], and felines [53]. However, the exact effect of FSH and LH on the resumption of meiosis during IVM remains unclear in horses, emphasizing the need for a more detailed revision of the concentrations and sources of hormones used in maturation media formulations and their effect in the equine COC.

Another group of important signaling molecules in the ovary belongs to the transforming growth factor beta (TGF-β) superfamily. It is well documented that these molecules are expressed by the oocyte and cumulus cells in numerous species and act as key intraovarian regulatory signals during the development of the follicle and oocyte [54]. One of these molecules is the anti-Müllerian hormone (AMH). In this study, the AMH gene (*AMH*) was upregulated in CC-MII samples compared to immature CC. It was also observed that the anti-Müllerian hormone receptor 2 gene (*AMHR2*) was expressed in OC and CC samples. These results suggest that AMH could have a paracrine role during equine oocyte maturation. A similar expression pattern of the anti-Müllerian gene was found in human CC and was positively associated with the developmental stage of the follicle, showing its highest expression in CC from pre-ovulatory follicles and observed within in vitro fertilization patients [55]. The anti-Müllerian hormone gene is expressed by granulosa cells in early follicle development, but this expression progressively changes to the CC as the follicle develops [56,57,58] due to the influence of paracrine factors secreted from the oocyte in a stage-specific type of regulation [58,59]. This supports the hypothesis that AMH participates in the crosstalk between oocyte and cumulus cells during maturation in horses. Interestingly, findings from studies in murine oocyte maturation showed that the supplementation of the maturation medium with recombinant AMH increased blastocyst formation [13]. However, when supplemented in the medium during IVM, it caused an inhibitory effect on FSH-induced cumulus expansion in mice, with a negative effect on the mRNA expressions of Hyaluronan synthase 2 (*Has2*), Pentraxin 3 (*Ptx3*), and TNF-alpha-induced protein 6 (*Tnfaip 6*) genes in COCs [60]. Thus, the exact signaling pathway and cascade events activated by AMH and its receptors, and their association with FSH activity in horse COCs, require further investigation. The results of the present study also show that the inhibin subunit α gene (*INHA*) is expressed in CC samples along with the inhibin subunit βA (*INHBA*). However, only *INHA* showed an increase in its expression in CC-MII samples. Inhibin is another TGF-β molecule that plays an endocrine role in the pituitary gland and a paracrine and autocrine role within the ovary and the follicle. Inhibin follows physiological cyclic activity in the mare, showing a peak in circulating concentrations at ovulation and the lowest concentration during the mid-luteal phase [61]. These results suggest the possibility that inhibin A participates through paracrine communication in the regulation of follicle development and possibly oocyte maturation. Another important TGF-β molecule participating in follicle development is the activin-binding protein follistatin (FST), which opposes the effect of activin and is, therefore, considered to have a role in promoting atresia or luteinization [54]. In the present study, *FST* expression was increased (log2FC: 4.25; FDR: 9.33 × 10^−10^) in CC-MII samples relative to immature CC. This observation suggests that FST has a role during the maturation of the oocyte and acquisition of developmental competence, as a higher abundance of FST mRNA has been associated with a faster time to the first cleavage in bovine zygotes [62]. Overall, this information highlights the importance of TGF-β molecules on the maturation of oocytes in horses.

An important event that occurs during the maturation of the COC is the expansion of the cumulus, which is the result of a series of modifications of the extracellular matrix (ECM) of cumulus cells surrounding the oocyte [63]. Cumulus expansion has been used as a marker for maturation success and blastocyst development [64]. During this process, hyaluronan is organized in a mesh-like network in the ECM and is stabilized by proteins and proteoglycans [65]. In this study, although the gene transcripts of the membrane-bound enzyme responsible for the synthesis of ECM hyaluronan (hyaluronan synthase, *HAS2*) were detected, no significant gene expression difference was found between immature CC and CC-MII. It has been proposed that the synthesis and accumulation of hyaluronan seems to be stimulated more potently by FSH rather than LH [66]. However, oocyte-secreted factors are still necessary to induce cumulus expansion [67]. It has been described in horses that the level of *HAS2* transcripts in mural granulosa cells can be transiently increased with human chorionic gonadotropin (hCG) treatment [68]. The lack of expression difference could suggest the inadequate content of gonadotropins in the maturation medium used in this study. Moreover, it emphasizes the importance of further investigating the effect of gonadotropin concentrations in maturation media.

Pathways related to extracellular space and the collagen trimer were significantly represented in the DEGs of CC samples. The analysis showed that multiple collagen genes had a lower expression in CC-MII. Collagens are trimeric molecules of the ECM that provide structural integrity to cells and tissues [69]. Collagens participate in the formation of the ECM due to their relatively high affinity to hyaluronan and integrins [65]. Currently, the importance of collagen synthesis in the ECM by CC is unknown in horses. In cattle, there is an increased deposition of laminin and type IV collagen in the intercellular space among CC during maturation, along with an increase in their expression during cumulus expansion [70]. However, the disruption of collagen during ovulation is a well-documented and necessary event [71]. It is important to consider that while the oocyte is maturing, the follicle prepares for ovulation, which implies changes in the extracellular space that require the proteolytic degradation of the ECM. Matrix metalloproteinases (MMP) are a family of endopeptidases with broad substrate specificity that are expressed in normal physiological situations and those that involve inflammation [72]. MMP9 is a 92 kDa gelatinase present in the basement membrane of the ECM; it plays an important role in basement membrane remodeling [73]. MMP1 is a collagenase that can cleave interstitial collagen and digest a number of other ECM and non-ECM molecules [74]. *MMP19* has been shown to be over-expressed in granulosa cells of large pre-ovulatory and ovulating follicles in mice [75]. Moreover, *MMP19* is downregulated in beta-estrogen receptor knockout (BERKO) mice that are known for presenting a common follicle rupture defect [76]. This suggests that the involvement of *MMP19* during follicular rupture is necessary for ovulation. In this study, we found an upregulation of *MMP1* and *MMP19* in OC-MII samples and a downregulation of *MMP9* and *MMP19* in CC-MII samples. It has been described that *MMP9* is only expressed in granulosa cells and not in cumulus cells in human COCs [77]. By contrast, the level of *MMP9* expression in bovine cumulus cells is correlated to the maturity level [78]. Although in vitro matured oocytes do not proceed through ovulation, it corresponds to a normal physiological event for which cumulus cells are programmed.

In conclusion, this study is the first report on gene expression changes in equine oocytes and cumulus cells during IVM. The data demonstrate changes in the transcript levels of LH and FSH receptors in CCs, as *LHCGR* expression is reduced while *FSHR* expression is increased in MII samples. Additionally, molecules of the TGF-β family seem to be important in the communication pathways of equine oocyte maturation. Genes associated with extracellular space, especially those related to the collagen trimer, show decreased expression in CC-MII samples, while there was no significant difference in the expression of hyaluronan synthesis genes. The information obtained in the present study may serve as a foundation to understand the dynamics of oocyte maturation and determine target modifications to the oocyte maturation system that could ultimately = increase the efficiency of IVP in horses.

## 4. Materials and Methods

### 4.1. Animals

Ten reproductively healthy mares of mixed and light breeds (Equus caballus) aged between 5 and 12 years were used. The mares were owned by the Rood & Riddle Equine Hospital, KY, USA, and were kept as part of a recipient embryo transfer herd. Mares were housed in paddocks with access to fresh water and hay ad libitum. Mares were used for only one transvaginal aspiration of oocytes (TVA) session during this study.

### 4.2. Collection of Cumulus–Oocyte Complexes

The TVA procedures were performed as described elsewhere [79] with the following modifications. Briefly, mares were restrained in stocks, the rectum was emptied, and the perineal area was aseptically cleaned. The sedation protocol included 0.02 mg/Kg i.v., butorphanol (Torbugesic^®^; Zoetis, Parsipanny, NJ, USA) and 0.01–0.02 mg/kg, i.v., detomidine (Dormosedan^®^; Zoetis, Parsipanny, NJ, USA). To facilitate manipulation, 0.16–0.24 mg/kg, i.v., N-butylscopolamine bromide (Buscopan^®^; Boehringer Ingelheim Vetmedica, Duluth, GA, USA) was administered to relax the smooth muscles of the rectum. Each mare was administered with 1 mg/kg, i.v., flunixin meglumine (Banamine^®^; Merck Animal Health, Madison, NJ, USA) and 6.6 mg/kg i.m. of ceftiofur crystalline free acid (Excede^®^; Zoetis, Parsipanny, NJ, USA) after the procedure. Once the mares were sedated, a customized-made vaginal extension with a micro convex probe (Exapad^®^ IMV Imaging, Rochester, MN, USA) was inserted into the vagina and antral follicles ranging from 5 to 25 mm in diameter were aspirated using a 60 cm double lumen needle (MILA International, Inc., Florence, KY, USA) connected to an aspiration pump (Pioneer Pro-Pump^®^, Pioneer Medical, Madison, CT, USA) and set at 150–200 mmHg. Each follicle was emptied and flushed 8 times using complete media (EMCARETM, ICP Bio Reproduction, Spring Valley, WI, USA) supplemented with heparin (5000 IU/mL). After all the follicles were aspirated, the collected content was filtered using a low-volume embryo filter (Em Con Embryo Transfer Filter, MAITM Animal Health, Elmwood, WI, USA), and the COCs were searched in a Petri dish under a stereoscope at room temperature. The recovered COCs were placed in an oocyte-holding medium (SyngroTM, Vetoquinol, Fort Worth, TX, USA) and kept at room temperature (22–23 °C) for 18 h.

### 4.3. Oocyte Maturation and Cumulus Cells Denuding

Only collected COCs with a compacted cumulus were used and randomly divided into two groups: immature and in vitro-matured. The immature group (*n* = 42) were placed into droplets of hyaluronidase (EQ-STRIPTM, IVF Bioscience, Manhattan, KS, USA) and cumulus cells were mechanically stripped from the oocytes by pipetting up and down several times using a micropipette (Cook Medical LLC, Bloomington, IN, USA) until all cells were detached and a completely clean zona pellucida could be observed. COCs assigned to the in vitro-matured group (*n* = 48) were rinsed and transferred to a 4-well plate with a CO_2_-equilibrated maturation medium (EQ-IVMTM, IVF Bioscience, Manhattan, KS, USA) following the manufacturer’s protocol. Briefly, COCs were incubated in humidified atmospheric air (21% O_2_, 6% CO_2_) at 38.2 °C for 24–26 h. After maturation, cumulus cells were removed from oocytes, as described, above and oocyte morphology was evaluated on denuded oocytes under a stereoscope to assess the perivitelline space, ooplasm, and zona pellucida. The maturity status was assessed by determining the presence of an extruded polar body [3].

Denuded oocytes and cumulus cells from immature and mature COCs were immediately transferred to individually labeled RNAase-free tubes, and 50 uL of RNALater^®^ (Thermo Fisher Scientific, Waltham, MA, USA) was added. Tubes were kept at room temperature for 20 min before being stored at −80 °C until the next step.

### 4.4. RNA Extraction

In a preliminary study, RNA was extracted from a single oocyte using the technique described below, resulting in an insufficient RNA yield for downstream analysis. Therefore, to obtain enough RNA material for sequencing, each sample unit was composed of a pool of six oocytes or cumulus cells from six COCs. This resulted in four groups described as follows: in vitro-matured CC (*n* = 3), immature CC (*n* = 3), in vitro-matured OC (*n* = 4), and immature OC (*n* = 4). RNA extraction was performed using the RNeasy Mini Kit #74104 (Qiagen, Germantown, MD, USA), followed by DNA digestion performed on-column using RNase-free DNase I #79254 (Qiagen, Germantown, MD, USA) according to the manufacturer’s guidelines. After extraction, RNA was quantified and assessed for integrity using the RNA 6000 Pico Assay Kit of the Bioanalyzer^®^ 2100 system (Agilent Technologies, Inc., Santa Clara, CA, USA).

### 4.5. Library Preparation, Next-Generation RNA Sequencing, and Bioinformatics Analysis

Extracted RNA was used for cDNA synthesis and mRNA library preparation using SMART-Seq^®^ v4 Ultra^®^ Low Input RNA Kit #634894 (Takara Bio, Inc., San Jose, CA, USA) and Nextra XT DNA library preparation Kit #FC-131-1024 (illumina, Inc., San Diego, CA, USA). RNA sequencing was conducted on the illumine NovaSeq 6000 (illumina, Inc., San Diego, CA, USA), generating, on average, 12 Gb/samples (150 bp paired-end reads). Reads were trimmed with TrimGalore 0.4.0 [80] for a quality check, and adapters were removed and then mapped to EquCab3.0 with the addition of the Y chromosome to the reference genome using STAR 2.7.2a. [81,82,83,84]. The mapped reads were quantified using featureCount [85]. Sequenced data are available using the sequence read archive accession number RJNA949261. Downstream analysis was performed using the DESeq2 method based on a false discovery rate (FDR) of 0.1 and a minimum fold change of 2.0, using the read counts [86]. To investigate the biological functions of DEGs, gene ontology analysis was performed using ShinyGO [87] and DAVID bioinformatics Resources version 6.8 [88]. We further investigated the potential crosstalk between oocytes and cumulus cells by matching DEGs from all samples to the available ligand–receptor pairs in the FANTOM5 database for protein-coding genes [89]. A graphic representation of the methodology pipeline flow in this study is presented in Supplementary Figure S1.

## Figures and Tables

**Table 1:** Pairs of ligand–receptors obtained from the FANTOM5 database and matched with DEG in OC and CC samples.

OC-Ligand	CC-Receptor	OC-Receptor	CC-Ligand
Tissue factor pathway inhibitor (*TFPI*) [up] ^1^	Coagulation factor III (*F3*) [up]	Activin A Receptor Type 1 (*ACVR1*) [up]	Anti-Mullerian Hormone (*AMH*) [up]
Glycoprotein hormones alpha chain (*CGA*) [up]	Follicle-stimulating hormone receptor (*FSHR*) [up]	Activin A Receptor Type 1 (*ACVR1*) [up]	Inhibin Subunit Alpha (*INHA*) [up]
Glycoprotein hormones alpha chain (*CGA*) [up]	Lutropin-choriogonadotropic hormone receptor (*LHCGR*) [down]	Protein Tyrosine Phosphatase Receptor Type Z1 (*PTPRZ1*) [down]	Midkine (*MDK*) [up]
Matrix metallopeptidase 1 (*MMP1*) [up]	CD44 antigen (*CD44*) [down]	Protein Tyrosine Phosphatase Receptor Type M (*PTPRM*) [down]	Cadherin 1 (*CDH1*) [down]
Semaphorin 4F (*SEMA4F*) [up]	Neuropilin 2 (NRP2) [down]		
Calmodulin 1 (*CALM1*) [up]	ATP binding cassette subfamily A member 1 (*ABCA1*) [down]		

^1^ Brackets indicate whether the genes were upregulated [up] or downregulated [down] in MII samples.

## Data Availability

Data generated during this study can be found in the manuscript and Supplementary files. Sequenced data are available at the National Center for Biotechnology Information (NCBI) with the sequence read archive accession number RJNA949261.

## Supplementary Materials

The following supporting information can be downloaded at: https://www.mdpi.com/article/10.3390/ijms241813718/s1.
