# Supplementary material for: Transcriptome Signature of Immature and In Vitro-Matured Equine Cumulus–Oocytes Complex
Source: Int J Mol Sci. 2023 Sep 6;24(18):13718. doi: 10.3390/ijms241813718 (PMC10531358; doi:10.3390/ijms241813718)
Supplement: Supplementary file 1 [file ijms-24-13718-s001.zip › Sup.Figure S1.pdf]

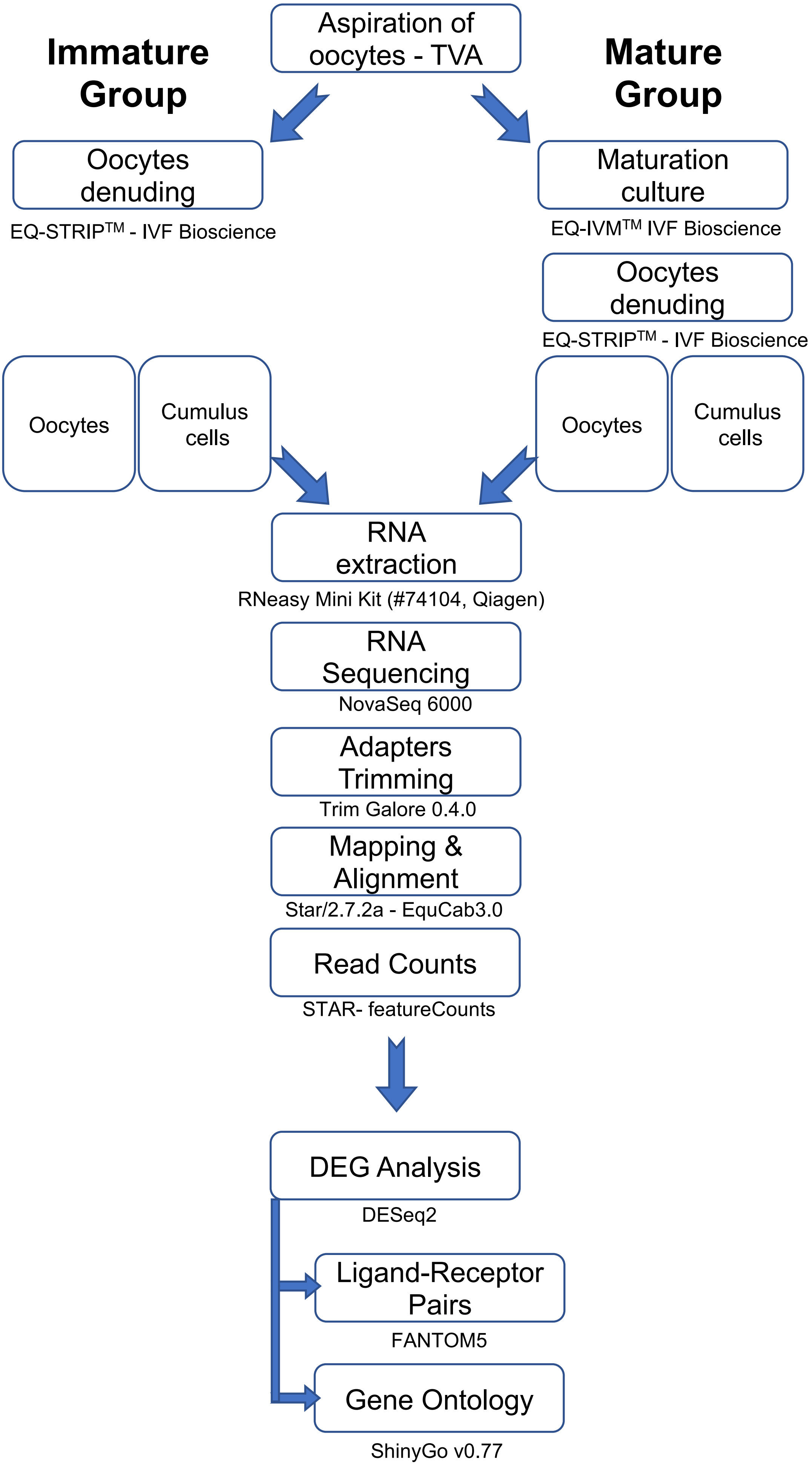

Figure S1. The pipeline detailing steps and the methodology used for sample acquiring, processing, and RNA work.
